# Genomic variant annotation workflow for clinical applications

**DOI:** 10.12688/f1000research.9357.2

**Published:** 2016-10-24

**Authors:** Thomas Thurnherr, Franziska Singer, Daniel J. Stekhoven, Niko Beerenwinkel

**Affiliations:** 1 Department of Biosystems Science and Engineering, ETH Zurich, Basel, Switzerland; 2 SIB Swiss Institute of Bioinformatics, Basel, Switzerland; 3 NEXUS Personalized Health Technologies, ETH Zurich, Zurich, Switzerland

**Keywords:** Drug-gene interaction, genomics, next-generation sequencing, annotation, somatic variant, clinical application, Bioconductor package, pipeline.

## Abstract

Annotation and interpretation of DNA aberrations identified through next-generation sequencing is becoming an increasingly important task. Even more so in the context of data analysis pipelines for medical applications, where genomic aberrations are associated with phenotypic and clinical features. Here we describe a workflow to identify potential gene targets in aberrated genes or pathways and their corresponding drugs. To this end, we provide the R/Bioconductor package rDGIdb, an R wrapper to query the drug-gene interaction database (DGIdb). DGIdb accumulates drug-gene interaction data from 15 different resources and allows filtering on different levels. The rDGIdb package makes these resources and tools available to R users. Moreover, rDGIdb queries can be automated through incorporation of the rDGIdb package into NGS sequencing pipelines.

## Introduction

In recent years, next-generation sequencing (NGS) pipelines have been established and employed extensively in research settings. These efforts have helped tremendously to improve our understanding of genetic malignancies such as cancer. More recently, joint efforts of research groups and clinics aim to further enhance our knowledge of these malignancies for better diagnostic and treatment options. For example, the Cancer Genome Atlas (TCGA) ^1^ Consortium has sequenced several thousand samples of more than 20 different cancer types. One of the aims of this project is to better characterize different cancer types, for example through identification of distinct molecular sub-types.

There are also substantial efforts to move NGS technologies and pipelines into molecular diagnostics, for example, for the characterization of somatic variants of individual tumor samples through targeted panel sequencing. Targeted panel sequencing covers a specific set of genes or locations, typically between 50 and a few hundred. Panels focus on frequently mutated or otherwise altered genes or genomic locations. Currently, several generic cancer panels and panels for specific cancer types are available ^2, 3^. Based on the panel characterization, targeted therapies for the specific genetic aberrations can be applied.

The number of targeted therapies for cancer available today is still relatively small and their approval is typically limited to one or several cancer sub-types ^4^. However, as the therapeutic options increase, more patients can benefit from these targeted therapies. As a consequence, several clinics or institutes developed and implemented molecular diagnostic approaches based on whole-exome and/or whole-genome sequencing ^5– 8^. Unlike targeted panels, whole-exome or whole-genome sequencing is not limited to a set of pre-selected genes, but allows for the detection of somatic aberrations across all protein coding sequences or the entire genome, respectively.

An exome- or genome-wide approach provides great advantage over targeted gene panels. They allow for a more comprehensive picture of the mutational landscape of a specific tumor. In addition, with more such data available and a better understanding of gene-gene and drug-gene interactions, prediction of drug efficacy as well as adverse drug reactions may become feasible. However, workflows based on whole-exome or whole-genome sequencing require clinical interpretation of the identified genetic variants. The result of an NGS pipeline is generally a list of genes harboring somatic variants or other genomic aberrations. To identify clinically actionable targets, these genomic aberrations need to be associated with drugs specifically targeting them.

Here we suggest a workflow to automate the identification of potential drug targets from a list of genomic aberrations, represented by a list of genes harboring them. For these genes, we mine drug-gene interactions using the drug-gene interaction database (DGIdb) ^9^. DGIdb integrates drug-gene interactions from 15 different resources. We provide the R/Bioconductor package rDGIdb ( http://bioconductor.org/packages/rDGIdb/), which allows to efficiently integrate drug-gene annotation with NGS pipelines. rDGIdb can query DGIdb and filter results on different levels, i.e., source databases, interaction types, and gene categories. Through the rDGIdb package, drug-gene interaction mining can be automated and incorporated easily into NGS pipelines. Moreover, the rDGIdb package also provides functionality to visualize results.

## Somatic variant calling

Somatic variants or other genomic aberrations are identified from raw sequencing data and filtered using a standard NGS pipeline. The number of somatic variants might vary substantially, depending on the sequencing approach used and the levels or stringency of filtering employed. Next, somatic variants are annotated with gene names, for which interacting drugs can then be queried through rDGIdb.

## Identification of targetable aberrations

Provided a list of genes with genomic aberrations, we identify aberrations targetable with a drug or compound. The R/Bioconductor package rDGIdb provides functionality to query drug-gene interactions provided by DGIdb and to apply filtering on different levels.

### R session setup

The package can be installed from an open R session. Instructions are provided on the rDGIdb Bioconductor page ( http://bioconductor.org/packages/rDGIdb/). After installation of the package and all its dependencies, rDGIdb needs to be attached and a gene vector prepared. Gene names can be loaded from a text file or manually entered. The code below illustrates how to load gene names from a text file called aberrated-genes.txt, assuming the text file lists one gene symbol per line.


                    library
                    (
                    "rDGIdb"
                    )

                    genes 
                    <- read
                    .
                    table
                    (
                    "aberrated-genes.txt"
                    , sep = 
                    "\t"
                    , header = FALSE, stringsAsFactors = FALSE)

                    genes 
                    <- 
                    genes[,1]


Alternatively, variants can be loaded from a variant call format (VCF) file and annotated using the Bioconductor VariantAnnotation workflow ^10^ ( http://bioconductor.org/packages/VariantAnnotation). This is illustrated in the rDGIdb package vignette.

### Query drug-gene interactions

To query DGIdb, the rDGIdb package provides a simple query function, queryDGIdb. The function takes a vector of official gene symbols for which drug-gene interactions are to be queried. This is the only required argument to the query function, all other arguments are optional.


                    genes 
                    <- c
                    (
                    "DDR2"
                    )

                    queryResult 
                    <- 
                    queryDGIdb(genes)
                

The function returns the query result as an object of type rDGIdbResult. The result is accessible through S4 methods. These methods format the result according to the result tabs provided on the DGIdb web interface. More specifically, the package provides four methods that return result data resembling the format provided through the DGIdb web interface, namely “Results Summary”, “Detailed Results”, “By Gene”, and “Search Term Summary”.


                    resultSummary(queryResult) 
                    # Summary table of the results

                    detailedResults(queryResult) 
                    # Detailed result table listing source and interaction type

                    byGene(queryResult) 
                    # Gene summary

                    searchTermSummary(queryResult) 
                    # Genes successfully mapped
                

**Figure 1.**
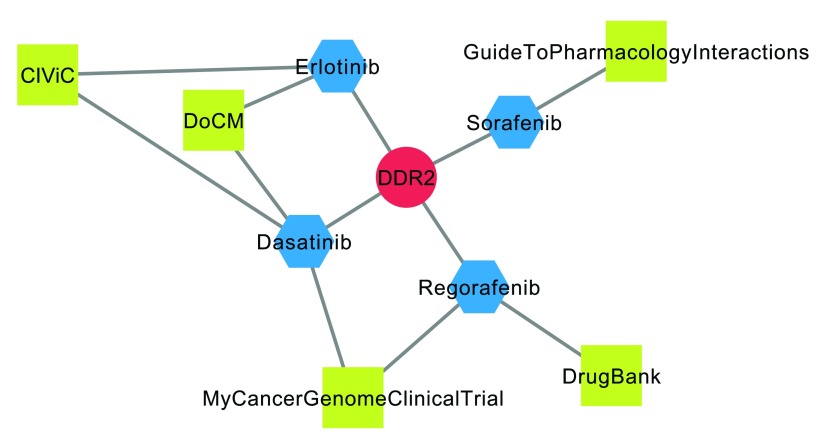
Drug-gene interactions illustrated as a network with *DDR2* in the middle (red) and interacting drugs (blue) connected to the gene. Resources that report a specific drug-gene interactions are colored in green.

An example output of resultSummary for the *DDR2* gene is shown in Table 1. Interactions are illustrated as a drug-gene interaction network in Figure 1. The figure further shows the resource that reported a specific interaction. Query results can either be further processed using R or saved to a text file for analysis with other software tools.

**Table 1.** rDGIdb result summary of *DDR2* drug interactions. The number in the table indicates if a drug-gene interaction was found in a source database, where 1 means yes and 0 means no. Drug-gene interactions are sorted by their score, which is the total number of source databases listing the interaction.

Gene	Drug	Drug-Bank	MyCancer- Genome- ClinicalTrial	GuideTo- Pharmacology- Interactions	CIViC	DoCM	Score
DDR2	DASATINIB	0	1	0	1	1	3
DDR2	ERLOTINIB	0	0	0	1	1	2
DDR2	REGORAFENIB	1	1	0	0	0	2
DDR2	SORAFENIB	0	0	1	0	0	1

## Filter drug-gene interactions

Depending on the application, it may be desirable to filter for specific drug-gene interactions. The rDGIdb package allows filtering on the level of (1) source database, (2) gene category, (3) interaction type, and (4) other criteria, applied directly to the query result.

### Filter by source database

DGIdb accumulates drug-gene interactions from 15 different source databases. These are summarized in Table 2. Depending on the application for which drug-gene interactions are queried, one or several source databases might be more relevant. The specific database or a group of databases to be queried is specified through the sourceDatabases argument. rDGIdb will only return hits listed in respective source databases. For example, the query below returns drug-gene interactions from databases: MyCancerGenome and MyCancerGenomeClinicalTrials only.


                    genes 
                    <- c
                    (
                    "KRAS"
                    , 
                    "BRAF"
                    )

                    databases 
                    <- c
                    (
                    "MyCancerGenome"
                    ,
                    "MyCancerGenomeClinicalTrials"
                    )

                    filter1 
                    <- 
                    queryDGIdb(genes, sourceDatabases = databases)
                

**Table 2.** Sources from which drug-gene interactions are accumulated in DGIdb.

Source	Link	Reference
CancerCommons	https://www.cancercommons.org	^11^
ChEMBL	https://www.ebi.ac.uk/chembl	^12^
CIViC	https://civic.genome.wustl.edu	^13^
ClearityFoundationBiomarkers	http://www.clearityfoundation.org	^14^
ClearityFoundationClinicalTrial	http://www.clearityfoundation.org/clinical-trials	^14^
DoCM	http://docm.genome.wustl.edu	^15^
DrugBank	http://www.drugbank.ca	^16^
GuideToPharmacologyInteractions	http://www.guidetopharmacology.org	^17^
MyCancerGenome	https://www.mycancergenome.org	^4^
MyCancerGenomeClinicalTrial	https://www.mycancergenome.org/clinicaltrials	^4^
PharmGKB	https://www.pharmgkb.org/	^18^
TALC	–	^19^
TEND	–	^20^
TdgClinicalTrial	–	^21^
TTD	http://bidd.nus.edu.sg/group/cjttd	^22^

The package provides a helper function that prints a list of all available source databases.


                    sourceDatabases()
                

### Filter by gene category

Similarly, we can filter for specific gene categories. With the gene categories filter, drug interactions for genes with a specific category label can be queried. Examples of gene categories are clinically actionable, kinase, or tumor suppressor. The optional geneCategories argument can be used to filter by gene categories.


                    categories 
                    <- c
                    (
                    "clinically actionable"
                    ,
                    "kinase"
                    , 
                    "tumor suppressor"
                    )

                    filter2 
                    <- 
                    queryDGIdb(genes, geneCategories = categories)
                

There are 41 different gene categories available. The following command lists all available gene categories.


                    geneCategories()
                

### Filter by interaction type

Finally, the package provides filtering by interaction type. An interaction type is a label for the type of drug-gene interaction. 33 different interaction types are available and examples are: activator, inhibitor, cofactor, or modulator. The code below illustrates how to filter for specific interaction types.


                    interactions 
                    <- c
                    (
                    "activator"
                    ,
                    "inhibitor"
                    )

                    filter3 
                    <- 
                    queryDGIdb(genes, interactionTypes = interactions)


To print a list of all available interaction types, one can use the following method:


                    interactionTypes()
                

### Manual filtering

Depending on the requirement of a specific application, additional filtering might be applied directly on the query results. For example, to increase confidence of results, drug-gene interactions might be filtered by setting a minimum cutoff on the score. As a result, only drug-gene interactions supported by a minimum number of source databases will be reported. Different score cutoffs may be employed, depending on whether the aim is to query interactions with support from multiple source databases or to include as many drug-gene interactions as there are available in the source databases. The example below illustrates how to filter out drug-gene interactions with only a single supporting source database from the result summary table.


                    subset
                    (resultSummary(filter2), Score > 1)
                

### Limitations of filtering

Although rDGIdb returns information on the type of interacting drug (such as inhibitor), to assist the follow-up interpretation of drug-gene interactions, querying and filtering through rDGIdb has limitations. For example, it is not possible to filter for specific drug-variant interactions. That is, variants in different locations of the same gene might have different biological effects in a cell or tumor. However, as querying is done on a gene level, variants can not be distinguished. Additional expert knowledge or other approaches will have to be employed to exclude non-relevant drug-gene interactions from the query results.

## Plotting of results

**Figure 2.**
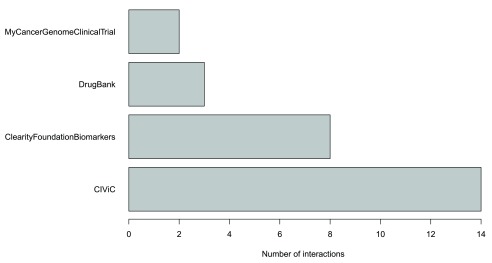
Example of the number of interactions by source for the KRAS gene.

The package allows basic plotting of the results. Specifically, the number of interactions by source database can be visualized. An example plot is provided in Figure 2. This plot indicates which source databases report specifically large or small number of drug-gene interactions.


                plotInteractionsBySource(filter2)
            

## Version numbers of DGIdb integrated resources

The rDGIdb package provides a function to print the version numbers of all resources integrated in DGIdb. This function helps users to decide if the resource versions available through rDGIdb are sufficient for their intended purpose.


                resourceVersions()
            

## Summary

We have described a workflow to identify potentially actionable genomic aberrations. More specifically, we have introduced the R/Bioconductor package rDGIdb, which provides an interface to query DGIdb using R. Given a list of genes with genomic aberrations, rDGIdb queries drug-gene interactions. The package allows filtering on different levels and visualization of the results. The rDGIdb package further includes detailed documentation and a vignette, which provides a step-by-step description of the workflow.

### Package content and dependencies

rDGIdb depends on jsonlite and httr, which are available in R version 3.3.1 or higher. Briefly, rDGIdb queries the API provided by DGIdb ( http://dgidb.genome.wustl.edu/api) using the POST function implemented in httr. Drug-gene interactions are returned by DGIdb in JSON format. Next, the data is deserialized into an R list object using the jsonlite package. Finally, the list is parsed and stored as an object of type rDGIdbResult. In order for rDGIdb to work, jsonlite, httr, and their dependencies need to be installed. A complete sessionInfo() output is provided below, which includes minimal version numbers of all dependencies.

• R version 3.3.1 (2016-06-21), x86_64-apple-darwin13.4.0
• Locale: en_US.UTF-8/en_US.UTF-8/en_US.UTF-8/C/en_US.UTF-8/en_US.UTF-8
• Base packages: base, datasets, graphics, grDevices, methods, stats, utils
• Other packages: rDGIdb 0.99.4
• Loaded via a namespace (and not attached): httr 1.1.0, jsonlite 1.0, R6 2.1.2, tools 3.3.1

## Software availability

1. Software available from: http://bioconductor.org/packages/rDGIdb/
2. Latest source code: https://github.com/Bioconductor-mirror/rDGIdb
3. Archived source code as at time of publication: http://dx.doi.org/10.5281/zenodo.59253 ^23^
4. License: MIT license

## References

[1] Cancer Genome Atlas Research Network: Comprehensive genomic characterization defines human glioblastoma genes and core pathways. *Nature.* 2008;455(7216):1061–1068. 10.1038/nature07385 18772890 PMC2671642

[2] Khodakov D Wang C Zhang DY: Diagnostics based on nucleic acid sequence variant profiling: PCR, hybridization, and NGS approaches. *Adv Drug Deliver Rev.* 2016;105(Pt A):3–19. 10.1016/j.addr.2016.04.005 27089811

[3] Easton DF Pharoah PD Antoniou AC: Gene-panel sequencing and the prediction of breast-cancer risk. *N Engl J Med.* 2015;372(23):2243–2257. 10.1056/NEJMsr1501341 26014596 PMC4610139

[4] Levy MA Lovly CM Pao W: Translating genomic information into clinical medicine: lung cancer as a paradigm. *Genome Res.* 2012;22(11):2101–2108. 10.1101/gr.131128.111 23019146 PMC3483539

[5] Clinical translation: NCT promotes swift translation of innovative high-throughput diagnostics into clinical practice. Accessed: 2016-06-22. Reference Source

[6] The Caryl and Israel Englander Institute for Precision Medicine at Weill Cornell Medical College. Accessed: 2016-06-22. Reference Source

[7] MD Anderson Cancer Center. Accessed: 2016-06-22. Reference Source

[8] Personalized medicine at the Mayo Clinic. Accessed: 2016-06-22. Reference Source

[9] Wagner AH Coffman AC Ainscough BJ: DGIdb 2.0: mining clinically relevant drug-gene interactions. *Nucleic Acids Res.* 2016;44(D1):D1036–D1044. 10.1093/nar/gkv1165 26531824 PMC4702839

[10] Obenchain V Lawrence M Carey V: VariantAnnotation: a Bioconductor package for exploration and annotation of genetic variants. *Bioinformatics.* 2014;30(14):2076–2078. 10.1093/bioinformatics/btu168 24681907 PMC4080743

[11] Shrager J Tenenbaum JM Travers M: Cancer Commons: Biomedicine in the internet age. In *Ekins/- Collaborative Computational Technologies for Biomedical Research.*Wiley-Blackwell;2011;161–177. 10.1002/9781118026038.ch11

[12] Bento AP Gaulton A Hersey A: The ChEMBL bioactivity database: an update. *Nucleic Acids Res.* 2014;42(Database issue):D1083–D1090. 10.1093/nar/gkt1031 24214965 PMC3965067

[13] CIViC: Clinical Interpretations of Variants in Cancer. Accessed: 2016-06-07. Reference Source

[14] The Clearity Foundation. Accessed: 2016-06-07. Reference Source

[15] DoCM: Database of Curated Mutations. Accessed: 2016-06-07. Reference Source 10.1038/nmeth.4000 PMC5317181 27684579

[16] Law V Knox C Djoumbou Y: DrugBank 4.0: shedding new light on drug metabolism. *Nucleic Acids Res.* 2014;42(Database issue):D1091–D1097. 10.1093/nar/gkt1068 24203711 PMC3965102

[17] Pawson AJ Sharman JL Benson HE: The IUPHAR/BPS Guide to PHARMACOLOGY: an expert-driven knowledgebase of drug targets and their ligands. *Nucleic Acids Res.* 2014;42(Database issue):D1098–D1106. 10.1093/nar/gkt1143 24234439 PMC3965070

[18] Whirl-Carrillo M McDonagh EM Hebert JM: Pharmacogenomics Knowledge for Personalized Medicine. *Clin Pharmacol Ther.* 2012;92(4):414–417. 10.1038/clpt.2012.96 22992668 PMC3660037

[19] Somaiah N Simon NG Simon GR: A tabulated summary of targeted and biologic therapies for non-small-cell lung cancer. *J Thorac Oncol.* 2012;7(16 Suppl 5):S342–S368. 10.1097/JTO.0b013e318271c798 23160320

[20] Rask-Andersen M Almén MS Schiöth HB: Trends in the exploitation of novel drug targets. *Nat Rev Drug Discov.* 2011;10(8):579–590. 10.1038/nrd3478 21804595

[21] Rask-Andersen M Masuram S Schiöth HB: The druggable genome: Evaluation of drug targets in clinical trials suggests major shifts in molecular class and indication. *Annu Rev Pharmacol Toxicol.* 2014;54(1):9–26. 10.1146/annurev-pharmtox-011613-135943 24016212

[22] Zhu F Han B Kumar P: Update of TTD: Therapeutic Target Database. *Nucleic Acids Res.* 2010;38(Database issue):D787–D791. 10.1093/nar/gkp1014 19933260 PMC2808971

[23] Thurnherr T: rDGIdb: First release [Data set]. *Zenodo.* 2016 Data Source

