# Supplementary material for: Genomic variant annotation workflow for clinical applications
Source: F1000Res. 2016 Oct 24;5:1963. Originally published 2016 Aug 12. [Version 2] doi: 10.12688/f1000research.9357.2 (PMC5130070; doi:10.12688/f1000research.9357.2)
Supplement: Peer review history

# Peer review: Referee response for version 2

**DOI:** 10.5256/f1000research.10559.r17155

**Published:** 2016-11-23

**Authors:** Ankush Sharma

**Affiliations:** 1 I​nstitute of Clinical Physiology, National Research Council, Siena, Italy

The authors have addressed the concerns raised by reviewers and made necessary changes in the manuscript. The manuscript is fully approved for indexing in F1000research.

I have read this submission. I believe that I have an appropriate level of expertise to confirm that it is of an acceptable scientific standard.

# Peer review: Referee response for version 2

**DOI:** 10.5256/f1000research.10559.r17154

**Published:** 2016-10-26

**Authors:** Christopher Southan

**Affiliations:** 1 IUPHAR/BPS Guide to PHARMACOLOGY, Center for Integrative Physiology, University of Edinburgh, Edinburgh, UK

Points raised were addressed in the revision

I have read this submission. I believe that I have an appropriate level of expertise to confirm that it is of an acceptable scientific standard.

# Peer review: Referee response for version 1

**DOI:** 10.5256/f1000research.10075.r15658

**Published:** 2016-09-13

**Authors:** Ankush Sharma, Md. Sahidul Islam

**Affiliations:** 1 I​nstitute of Clinical Physiology, National Research Council, Siena, Italy; 2 Department of Statistics, University of Rajshahi, Rajshahi, Bangladesh

This R Package "rDGidb" is of immense usability for genomics and proteomics research community for integrating drug interactions with variations obtained from NGS data and researchers studying complex multi target drug-gene/protein interactions. The research article is written clearly and well organized except for results section which has a room for improvement.

 The minor concerns are outlined as follows:- 

1. We recommend authors to demonstrate results shown in Table 1 as a pictorial representation such as drug-gene interaction network to increase readability.
2. We suggest inclusion of the information related to Source Trust Level.
3. It would be nice to include query option using **r**eference **S**NP ID number (“ **rs**” ID) or by chromosomal position of genomic aberrations obtained from Next Generation Sequencing pipeline to directly identify drugs associated with these clinically actionable variations.   

We encountered a problem in installation of package “rDGidb”in R (version 3.3.1, release date 2016-06-21) with a warning message i.e. Package ‘rDGIdb’ is not available (for R version3.3.1).  

 We recommend authors to make “rDGidb” working and if this warning message is platform dependent, then please provide detailed documentation on software’s or any updates needed in existing packages before installation of package.

We have read this submission. We believe that we have an appropriate level of expertise to confirm that it is of an acceptable scientific standard, however we have significant reservations, as outlined above.

# Response

**Published:** 2016-10-10

**Authors:** Thomas Thurnherr

We would like to thank Ankush Sharma and Sahidul Islam for their time and effort to review our manuscript. The concerns are addressed below:

1. As suggested, we added a figure to the manuscript that illustrates drug-gene interactions of *DDR2.*
2. “Source trust level” is a parameter available through the DGIdb API, but not through the DGIdb web interface. The parameter can either be set to “expert curated” or “non-curated”. We did not include it as a parameter in rDGIdb for mainly two reasons: 1) We aim to reflect the web interface as consistently as possible and “Source trust level” is not available for DGIdb website queries; and 2) It is not clear which resources or drug-gene interactions are considered “expert curated” and which are not.
3. Thank you for the suggestion. Other packages implement variant call format (VCF) file import and annotation functionality. We recommend to use those. However, we added a paragraph to the manuscript (section “R session setup”) on that topic. Moreover, in the package vignette, we show how to employ the workflow with a VCF file as input. With this, we now provide a complete annotation workflow, from variants in VCF format to drug-gene interactions. Finally, we would like to point out that DGIdb queries are currently only possible on a gene level, but not on a variant level. Therefore, the association of a mutation in a specific position of the genome with a drug requires manual curation of the results obtained through rDGIdb.

Finally, the package is not yet available in the current Bioconductor release branch (version 3.3). This is likely the reason why you encountered an error while installing the package. The release is scheduled for October 2016 (version 3.4). The release will make the package available through the standard installation procedure. Installation instructions for packages in the development branch are provided on the Bioconductor website (https://www.bioconductor.org/developers/).

# Peer review: Referee response for version 1

**DOI:** 10.5256/f1000research.10075.r15657

**Published:** 2016-08-18

**Authors:** Christopher Southan

**Affiliations:** 1 IUPHAR/BPS Guide to PHARMACOLOGY, Center for Integrative Physiology, University of Edinburgh, Edinburgh, UK

This describes an R-based tool to query the drug-gene interactions in DGIdb. The paper is well written and the tool clearly has some utility. However, my reservations are outlined below.

1. As the application of NGS to cancer samples accelerates the resultant explosion of somatic variants threatens to swamp user’s ability to select them to input to this tool. What filters can be put in place to reduce huge aberration lists associated with passenger (i.e. probably non-causative and spurious) rather than driver mutations?
2. Given the latest Nature publication on the analysis of protein-coding genetic variation in 60,706 humans now available in the ExAC resource I suggest the utility emphasis for looking at germ-line vs somatic target aberrations should be re-balanced.
3. According to their website,  DGIdb (v2.22 - sha1 aa9170e) was last updated 2016-02-21 and not all primary sources loaded were the latest versions even then.  For example DrugBank is now up to 5.0 and GtoPdb is up to 2016.3 and it is not clear if it has only ChEMBL 20 rather than 21. Unless DGIdb can be prevailed upon to update more frequently and provide the release statistics of content, the utility of this tool is constrained because users cannot trust the results to be up to date.
4. The main goal of this tool for the identification of targetable aberrations will be confounded by the conflation of loss vs gain of function on both the target and drug sides.  As we know, genetic aberrations are predominantly LOF but most drugs also negatively modulate their targets. This should be discussed and perhaps even made filterable in some way?
5. Why does Table 1 show such an apparently inconsistent mosaic of results? Reasons for discordance between the individual sources need to be explained.
6. Given this tool was developed by SIB would it be possible to add in Swiss-Var as an independent source via Swiss-Prot or NeXtProt?

I have read this submission. I believe that I have an appropriate level of expertise to confirm that it is of an acceptable scientific standard, however I have significant reservations, as outlined above.

# Response

**Published:** 2016-10-10

**Authors:** Thomas Thurnherr

We would like to thank Christoper Southan for his thoughtful comments. Please find our response below:

1. There are several strategies that can be put in place to reduce the number of somatic aberrations to those which are likely relevant/functional. These include, but are not limited to: 1) Identification of recurrent mutations; 2) Prediction of the functional impact of a mutation; 3) Identification of recurrent combinations of mutations; and 4) Experimental validation. At this point, we would like to remark that the aim of rDGIdb is to annotate variants with potential drug-gene interactions and not to filter somatic variants. Other methods take care of filtering variants. Finally, rDGIdb is not limited in the number of genes to query. We query drug-gene interactions for several thousand genes at the time.
2. We agree that germline mutations play a role in a variety of diseases, specifically in cancer. Although rDGIdb is not limited to somatic mutations, we aim to identify potential targeted therapies. That is, drugs that specifically target malignant cells. Therefore, we think that considering somatic mutations rather than germline mutations is justified in this case.
3. We agree with the reviewer that DGIdb does not currently use the latest versions of all the resources it integrates. As a consequence, drug-gene interactions queried through rDGIdb might not agree with results from the most up-to-date resources. Results queried through rDGIdb are based on results from DGIdb and the resources it integrates. As a consequence, we have no control over how frequently resources are updated by DGIdb. However, we added a function to the package that prints the versions of all resource integrated by DGIdb. This helps the user to decide if the version available in rDGIdb/DGIdb is sufficient for the intended purpose. The function is documented in the updated manuscript and in the package vignette.
4. The type of a drug-gene interaction can be filtered through an optional argument (interactionType) to the main query function. Possible values include suppressor, inhibitor, or activator. These limit reported drug-gene interactions to the interaction type of interest. Moreover, rDGIdb allows to query for specific gene categories, for instance tumor suppressor. These information/filters may help the user interpret the results provided by rDGIdb. Finally, we would like to point out that the interpretation of mutations in regard of their suitability as targetable mutations is beyond the scope of rDGIdb. All described filters and additional information can only assist the user in the interpretation of a specific mutation or interaction. The applicability of a certain therapy depends on a number of factors: cancer type, treatment history, and many others.
5. We selected an example that is brief enough to be presented as a table in the manuscript. *DDR2* seemed reasonable, with drug interactions in five different resources. The diversity of the drug-gene interactions in Table 1 can be explained by the diversity of these resources. For example, DrugBank lists experimental and approved drugs in any disease. In contrast, MyCancerGenomeClinicalTrials and CIViC list drugs in cancer only, which have either been approved by the authorities or are currently investigated through a clinical study. Finally, in the manuscript we mention that the most appropriate resources to be queried might depend on the application. We further explain how to filter for specific resources.
6. To our knowledge, SwissVar does not catalogue drug-gene interactions, but provides information on variants and their disease relations. At the moment, the main focus of the package is to report drug-gene interactions. However, we agree that SwissVar provides useful additional information on the genes queried through rDGIdb. Therefore, we consider an extension of the scope in regard to disease associations for a future release of the package.
